# Correction: A 10+10+30 radio campaign is associated with increased infant vaccination and decreased morbidity in Jimma Zone, Ethiopia: A prospective, quasi-experimental trial

**DOI:** 10.1371/journal.pgph.0001994

**Published:** 2023-05-25

**Authors:** Bernard Appiah, Lakew Abebe Gebretsadik, Abebe Mamo, Brittany Kmush, Yisalemush Asefa, Christopher R. France, Elfreda Samman, Tena Alemayehu, Mahdiya Abafogi, Md Koushik Ahmed, Laura Forastiere, Gursimar Kaur Singh, David Larsen, Sudhakar Morankar

## Notice of republication

This article was republished on May 10, 2023, to include an author who was inadvertently omitted from the byline in the final stages before publication. Please download this article again to view the correct version. The originally published, uncorrected article and the republished, corrected articles are provided here for reference.[1]

## Supporting information

S1 File Originally published, uncorrected article. (PDF)

S2 File Republished, corrected article. (PDF)
