# Supplementary material for: A 10+10+30 radio campaign is associated with increased infant vaccination and decreased morbidity in Jimma Zone, Ethiopia: A prospective, quasi-experimental trial
Source: PLOS Glob Public Health. 2022 Nov 2;2(11):e0001002. doi: 10.1371/journal.pgph.0001002 (PMC10021526; doi:10.1371/journal.pgph.0001002)
Supplement: S1 Table — (DOCX) [file pgph.0001002.s001.docx]

**S1 Table: Baseline characteristics of the participants in the per protocol analysis**

| Characteristic | Control  (N (%))  N=302 | Intervention  (N (%))  N=108 | X^2^ p-value |
| --- | --- | --- | --- |
| Sex of the child |  |  | 0.64 |
| boy | 157 (52.0%) | 59 (54.6%) |  |
| girl | 145 (48.0%) | 49 (45.4%) |  |
| Birth order |  |  | 0.15 |
| 1st | 75 (24.8%) | 34 (31.5%) |  |
| 2nd-3rd | 129 (42.7%) | 49 (45.4%) |  |
| 4+ | 98 (32.5%) | 25 (23.1%) |  |
| Education |  |  | <0.001 |
| No formal education | 145 (48.5%) | 29 (26.9%) |  |
| Primary | 94 (31.4%) | 52 (48.1%) |  |
| Secondary or higher | 60 (20.1%) | 27 (25.0%) |  |
| Religion |  |  | 0.005 |
| Islam | 213 (70.5%) | 93 (86.1%) |  |
| Orthodox | 56 (18.5%) | 8 (7.4%) |  |
| Other | 33 (10.9%) | 7 (6.5%) |  |
| Marital Status |  |  | 0.19 |
| Married | 299 (99.0%) | 105 (97.2%) |  |
| Other | 3 (1.0%) | 3 (2.8%) |  |
| Residence |  |  | 0.28 |
| Rural | 274 (90.7%) | 94 (87.0%) |  |
| Urban | 28 (9.3%) | 14 (13.0%) |  |
| Time to vaccination site |  |  | 0.036 |
| < 15 Minutes | 58 (19.2%) | 33 (30.6%) |  |
| 15-30 Minutes | 139 (46.0%) | 47 (43.5%) |  |
| > 30 Minutes | 105 (34.8%) | 28 (25.9%) |  |
| Place of Birth |  |  | 0.065 |
| Yours, relative's, or neighbour's home | 83 (27.5%) | 20 (18.5%) |  |
| Hospital, Health Center, or Health Post | 219 (72.5%) | 88 (81.5%) |  |
| Frequency of ANC |  |  | 0.043 |
| No | 39 (12.9%) | 5 (4.6%) |  |
| 1-3 | 162 (53.6%) | 59 (54.6%) |  |
| 4+ | 101 (33.4%) | 44 (40.7%) |  |
| Card Verified |  |  | <0.001 |
| Verified | 216 (71.5%) | 41 (38.0%) |  |
| Not Verified | 86 (28.5%) | 67 (62.0%) |  |
| Household Radio Ownership | 131 (43.4%) | 65 (60.2%) | 0.003 |
| Frequency of Listening to the Radio |  |  | <0.001 |
| Almost every day | 10 (3.3%) | 25 (23.1%) |  |
| At least once a week | 8 (2.6%) | 30 (27.8%) |  |
| Less than once a week | 17 (5.6%) | 20 (18.5%) |  |
| Not at all | 267 (88.4%) | 33 (30.6%) |  |
| Household Mobile Phone Ownership | 134 (44.4%) | 80 (74.1%) | <0.001 |
|  | **Mean (SD)** | **Mean (SD)** | **t-test p-value** |
| Age of the Child (days) | 16.51 (10.53) | 17.40 (11.63) | 0.47 |
| Age of mother (years) | 26.73 (6.07) | 25.26 (5.23) | 0.026 |
| Wealth Index | 6.44 (2.14) | 7.44 (3.62) | <0.001 |
