# Supplementary material for: A 10+10+30 radio campaign is associated with increased infant vaccination and decreased morbidity in Jimma Zone, Ethiopia: A prospective, quasi-experimental trial
Source: PLOS Glob Public Health. 2022 Nov 2;2(11):e0001002. doi: 10.1371/journal.pgph.0001002 (PMC10021526; doi:10.1371/journal.pgph.0001002)
Supplement: S2 Table — (DOCX) [file pgph.0001002.s002.docx]

**S2 Table: Vaccination outcomes in the control and intervention groups^a^**

|  | Intention to Treat | | | | Per-Protocol | | |  |
| --- | --- | --- | --- | --- | --- | --- | --- | --- |
| **Vaccine** | **Control**  **(N (%))**  **N=324** | **Intervention**  **(N (%))**  **N=314** | **X^2^**  **p-value** | **Control**  **(N (%))**  **N=302** | | **Intervention**  **(N (%))**  **N=108** | **X^2^**  **p-value** | |
| Penta3 | 92 (28.4%) | 173 (55.1%) | <0.001 | 90 (29.8%) | | 76 (70.4%) | <0.001 | |
| Penta2 | 171 (52.8%) | 207 (65.9%) | <0.001 | 151 (50.0%) | | 88 (81.5%) | <0.001 | |
| Penta1 | 187 (57.7%) | 239 (76.1%) | <0.001 | 166 (55.0%) | | 94 (87.0%) | <0.001 | |
| Rota2 | 173 (53.4%) | 208 (66.2%) | <0.001 | 153 (50.7%) | | 88 (81.5%) | <0.001 | |
| Rota1 | 183 (56.5%) | 238 (75.8%) | <0.001 | 162 (53.6%) | | 94 (87.0%) | <0.001 | |
| PCV3 | 91 (28.1%) | 171 (54.5%) | <0.001 | 89 (29.5%) | | 74 (68.5%) | <0.001 | |
| PCV2 | 167 (51.5%) | 206 (65.6%) | <0.001 | 147 (48.7%) | | 88 (81.5%) | <0.001 | |
| PCV1 | 183 (56.5%) | 239 (76.1%) | <0.001 | 162 (53.6%) | | 94 (87.0%) | <0.001 | |
| OPV3 | 26 (8.0%) | 191 (60.8%) | <0.001 | 26 (8.6%) | | 85 (78.7%) | <0.001 | |
| OPV2 | 131 (40.4%) | 250 (79.6%) | <0.001 | 111 (36.8%) | | 100 (92.6%) | <0.001 | |
| OPV1 | 123 (38.0%) | 267 (85.0%) | <0.001 | 102 (33.8%) | | 106 (98.1%) | <0.001 | |
| Fully Vaccinated^b^ | 24 (7.4%) | 139 (44.3%) | <0.001 | 24 (7.9%) | | 68 (63.0%) | <0.001 | |

^a^Only those with vaccine dates recorded on a health card were counted as vaccinated.

^b^Fully vaccinated defined as receiving all doses of Penta, Rota, PCV, and OPV (excluding birth dose) with dates recorded on the health card.
