# Supplementary material for: A 10+10+30 radio campaign is associated with increased infant vaccination and decreased morbidity in Jimma Zone, Ethiopia: A prospective, quasi-experimental trial
Source: PLOS Glob Public Health. 2022 Nov 2;2(11):e0001002. doi: 10.1371/journal.pgph.0001002 (PMC10021526; doi:10.1371/journal.pgph.0001002)
Supplement: S3 Table — (DOCX) [file pgph.0001002.s003.docx]

**S3 Table: Regression analysis for vaccination outcomes comparing the intervention to control groups^a^**

|  | Intention to Treat (N=638) | | | | | | Per protocol (n=410) | | | | | |
| --- | --- | --- | --- | --- | --- | --- | --- | --- | --- | --- | --- | --- |
| **Outcome** | **Un-adjusted RR** | **95% CI** | **P-value** | **Adjusted RR^c^** | **95% CI** | **P-Value** | **Un-adjusted RR** | **95% CI** | **P-value** | **Adjusted RR^c^** | **95% CI** | **P-Value** |
| Penta 3 | 1.95 | 1.58-2.40 | <0.000 | 1.96 | 1.57-2.45 | <0.000 | 2.36 | 1.86-3.00 | <0.000 | 2.66 | 2.09-3.39 | <0.000 |
| Penta 2 | 1.26 | 1.08-1.47 | 0.003 | 1.28 | 1.09-1.51 | 0.003 | 1.63 | 1.39-1.91 | <0.000 | 1.73 | 1.44-2.07 | <0.000 |
| Penta 1 | 1.32 | 1.18-1.48 | <0.000 | 1.26 | 1.10-1.46 | <0.001 | 1.57 | 1.39-1.77 | <0.000 | 1.56 | 1.32-1.85 | <0.000 |
| Rota 2 | 1.25 | 1.08-1.45 | 0.003 | 1.26 | 1.07-1.48 | 0.005 | 1.60 | 1.37-1.87 | <0.000 | 1.68 | 1.41-2.03 | <0.000 |
| Rota 1 | 1.34 | 1.19-1.52 | <0.000 | 1.29 | 1.11-1.48 | <0.001 | 1.61 | 1.42-1.83 | <0.000 | 1.62 | 1.36-1.94 | <0.000 |
| PCV 3 | 1.95 | 1.58-2.40 | <0.000 | 2.02 | 1.61-2.52 | <0.000 | 2.32 | 1.81-2.98 | <0.000 | 2.68 | 2.09-3.43 | <0.000 |
| PCV 2 | 1.29 | 1.10-1.51 | 0.002 | 1.31 | 1.10-1.56 | <0.003 | 1.69 | 1.42-1.99 | <0.000 | 1.79 | 1.46-2.18 | <0.000 |
| PCV 1 | 1.35 | 1.20-1.52 | <0.000 | 1.29 | 1.12-1.49 | <0.000 | 1.61 | 1.41-1.82 | <0.000 | 1.59 | 1.34-1.89 | <0.000 |
| OPV 3 | 8.04 | 5.07-12.7 | <0.000 | 8.54 | 5.45-13.3 | <0.000 | 9.51 | 6.02-15.0 | <0.000 | 11.1 | 7.38-16.8 | <0.000 |
| OPV 2 | 1.95 | 1.74-2.19 | <0.000 | 2.07 | 1.81-2.38 | <0.000 | 2.72* | 2.44-3.04 | <0.000 | 2.90* | 2.43-3.46 | <0.000 |
| OPV 1 | 2.16* | 1.99-2.34 | <0.000 | 2.27* | 1.92-2.55 | <0.000 | 3.01* | 2.88-3.15 | <0.000 | 3.20* | 2.64-3.88 | <0.000 |
| Fully Vaccinated^b^ | 6.18 | 3.86-9.90 | <0.000 | 6.49 | 4.10-10.0 | <0.000 | 9.44 | 5.72-15.6 | <0.000 | 10.5 | 6.60-16.7 | <0.000 |
| ^a^Only those with vaccine dates recorded on a health card were counted as vaccinated.  ^b^ Fully vaccinated defined as receiving all doses of Penta, Rota, PCV, and OPV (excluding birth dose).  ^c^Adjusted for sex of child, birth order, religion, marital status, urban/rural, time to vaccination centre, place of delivery, antenatal care, able to see age/vaccination verification card, age of child in days at baseline, age of mother in years, wealth index, radio listening, and accounting for clustering within kebeles. Unadjusted models still accounted for clustering within kebeles.  *Convergence not achieved  RR: Risk Ratio  CI: Confidence Interval | | | | | | | | | | | | |
