# Supplementary material for: A 10+10+30 radio campaign is associated with increased infant vaccination and decreased morbidity in Jimma Zone, Ethiopia: A prospective, quasi-experimental trial
Source: PLOS Glob Public Health. 2022 Nov 2;2(11):e0001002. doi: 10.1371/journal.pgph.0001002 (PMC10021526; doi:10.1371/journal.pgph.0001002)
Supplement: S1 File — (DOCX) [file pgph.0001002.s004.docx]

**Development and implementation of 10+10+30 Radio campaign on child vaccination in Ethiopia**

**Detailed Intervention Development:** During the planning phase, an inception workshop was held on November 27, 2018 to provide relevant information on the project to key stakeholders, including Jimma University (JU) officials, key community radio station staff and health extension workers (HEWs). The workshop was followed by focus group discussions (FGDs) with HEWs, mothers with infants and radio actors in May 2019 to gather participant perspectives on opportunities and challenges of using radio programming to promote infant vaccination.

A four-day drama design workshop was conducted to identify, among other items, the title for the serial drama, the number of episodes that should be produced, as well as the duration and sequence of each episode. The team also used the workshop to identify the measurable objectives, purpose, and precise message content for each episode. The workshop drew 18 participants who were affiliated with institutions such as the Federal Ministry of Health, Ethiopia; UNICEF Ethiopia; Ethiopian Broadcasting Corporation; Jimma University, and Jimma Community Radio. The outcome of the radio drama design workshop was used to create scripts for the drama. After discussing nine titles for the radio intervention, Egeree Ijoollee (Child’s Future) was selected by combining multiple suggested names. Women/caregivers of infants were identified as the primary audience, husbands or household heads as secondary audiences, and health workers and community leaders and social networks as tertiary audiences.

**Training for community health workers:** In February 2020, 13 community extension workers participated in a two-day workshop including role playing techniques to build their skills as radio panellists. The workshop covered topics such as how to prepare to become an effective panellist on radio, and how to answer questions from a facilitator and listeners.

**Radio campaign implementation:** During the campaign’s implementation on radio, the 10-minute pre-recorded serial drama was aired. Immediately after the drama episode, a trained journalist acting as a facilitator asked questions on the drama episodes for two trained health extension workers (HEWs) acting as radio panellists to answer. After the discussion, the radio station’s phone line was opened for listeners to ask questions or comment on the programme live. For those who were unable to call in, they could also text messages, which were read by the facilitator for the two trained HEWs to answer. The HEWs and the facilitator were compensated for their time. Each HEW received 1,200 Ethiopian birrs (about $24.6) per day of the radio broadcast to cater for both transportation and their time, while the facilitator received 600 Ethiopian birrs (about $12.30) for their time. The program was aired on Jimma Community Radio from October 23, 2020 through January 10, 2021. The episodes aired each week on Fridays and Sundays at 2:00 pm.

Listeners called or texted to express their appreciation for the programme while others asked questions including which vaccines are given at different health facilities, which diseases vaccines can prevent, how the diseases spread, and why more attention are being given to children. In other words, the HEWS did not already have questions from listeners during the 30 minutes session. Instead, the questions were posed live but the facilitator who determined the order of questions to be answered. Listening to the radio programme was a community event for some listeners, especially during the coffee ceremony.
