# Supplementary material for: Correction: A 10+10+30 radio campaign is associated with increased infant vaccination and decreased morbidity in Jimma Zone, Ethiopia: A prospective, quasi-experimental trial
Source: PLOS Glob Public Health. 2023 May 25;3(5):e0001994. doi: 10.1371/journal.pgph.0001994 (PMC10212110; doi:10.1371/journal.pgph.0001994)
Supplement: S1 File — (PDF) [file pgph.0001994.s001.pdf]

PLOS GLOBAL PUBLIC HEALTH

RESEARCH ARTICLE

A 10+10+30 radio campaign is associated with increased infant vaccination and decreased morbidity in Jimma Zone, Ethiopia: A prospective, quasi-experimental trial

Bernard Appiah[1,2]*, Lakew Abebe Gebretsadik[3], Abebe Mamo[3], Brittany Kmush[4], Yisalemush Asefa[5], Christopher R. France[6], Elfreda Samman[7], Tena Alemayehu[8], Mahdiya Abafogi[9], Md Koushik Ahmed[1], Laura Forastiere[9], Gursimar Kaur Singh[10], David Larsen[4]

1 Department of Public Health, Research Program on Health Communication and Public Engagement (H-COPE), Syracuse University, Syracuse, New York, United States of America, **2** Centre for Science and Health Communication, Accra, Ghana, **3** Department of Health, Behaviour & Society, Jimma University, Jimma Town, Ethiopia, **4** Department of Public Health, Syracuse University, Syracuse, New York, United States of America, **5** Department of Health Management & Policy, Jimma University, Jimma Town, Ethiopia, **6** Department of Psychology, Ohio University, Athens, Ohio, United States of America, **7** Department of Health Promotion and Community Health Sciences, Texas A&M University School of Public Health, College Station, Texas, United States of America, **8** Department of Theatre Arts, Jimma University, Jimma Town, Ethiopia, **9** Jimma Zone Health Office, Jimma Town, Ethiopia, **10** Department of Biostatistics, Yale School of Public Health, New Haven, Connecticut, United States of America

* beappiah@syr.edu

OPEN ACCESS

Citation: Appiah B, Gebretsadik LA, Mamo A, Kmush B, Asefa Y, France CR, et al. (2022) A 10+10+30 radio campaign is associated with increased infant vaccination and decreased morbidity in Jimma Zone, Ethiopia: A prospective, quasi-experimental trial. PLOS Glob Public Health 2(11): e0001002. https://doi.org/10.1371/journal.pgph.0001002

Editor: Hanna Nalecz, Instytut Matki i Dziecka, POLAND

Received: March 23, 2022

Accepted: October 3, 2022

Published: November 2, 2022

Copyright: © 2022 Appiah et al. This is an open access article distributed under the terms of the Creative Commons Attribution License, which permits unrestricted use, distribution, and reproduction in any medium, provided the original author and source are credited.

Data Availability Statement: The dataset that supports the findings of this study is available in Figshare with the identifier given below: https://doi.org/10.6084/m9.figshare.20309730.v1.

Funding: This work was funded by the Bill & Melinda Gates Foundation (grant number OPP1191306). The grant was awarded to BA as the principal investigator. The funder of the study did

Abstract

Mass media interventions have the potential to reach large audiences and influence health behaviours and outcomes. To date, no study has evaluated the effect of a radio-only campaign on infant vaccination coverage, timeliness, and related morbidity in a low-income country. We implemented the "10+10+30" radio campaign involving broadcasting a weekly 10-minute radio drama series on vaccination, followed by a 10-minute discussion by community health workers, and then a 30-minute listener phone-in segment in Jimma Zone, Ethiopia for three months. To assess the impact of 10+10+30, which was aired on a community radio station, we recruited mothers of infants up to 5 weeks old in intervention district clusters that were inside the radio station's reception range (n = 328 dyads) and control district clusters that were outside of the range (n = 332 dyads). Intention-to-treat and per-protocol analyses, adjusted for pre-intervention differences between the districts, were conducted to examine the co-primary outcome of Penta-3 vaccination coverage and timeliness as well as those of other vaccines and outcomes related to infant morbidity. Both intention-to-treat and per-protocol analyses revealed higher vaccine coverage (p<0.001) and more timely vaccine administration (p<0.001) in the intervention district relative to the control district, with infants in the intervention district being 39% more likely to receive a Penta 3 vaccination (adjusted RR: 1.39, p<0.001). In addition, adjusted regression analyses of maternal retrospective reports over a two-week period revealed 80% less infant diarrhoea (RR: 0.20, p<0.001), 40% less fever (RR: 0.60, p<0.001) and 58% less cough (RR: 0.42, p<0.001) in the intervention district relative to the control district. This study provides

not play any role designing the study, data analysis, data interpretation, or reporting of the results.

Competing interests: The authors have declared that no competing interests exist.

compelling initial evidence that a radio drama integrated with discussion and phone-in components may improve infant vaccination coverage and timeliness, and may reduce infant morbidity. Randomized controlled trials are needed to confirm and extend these findings with other samples.

Introduction

Coverage and timeliness of childhood vaccination are critical global health challenges, particularly in low- and middle-income countries. Childhood vaccination effectively avoids negative health outcomes and death related to vaccine-preventable diseases. A recent study estimated that the return on investment associated with achieving expected coverage of vaccines that target ten antigens in 94 low- and middle-income countries was about 16 times greater than costs during 2011–2020 [1].

Given the enormous potential health and economic benefits of adequate vaccine coverage, governments and other key stakeholders have invested in diverse interventions for promoting childhood vaccination [2]. Current interventions include health facility-based education, community-based health education strategies (e.g., mass media campaigns), and incentives for vaccination providers [3]. Whereas mass media interventions to improve childhood vaccination have received some attention in low- and middle-income countries [4], those that focus specifically on radio are rare. For example, a Cochrane systematic review of interventions to improve infant vaccination in low- and middle-income countries (LMICs) identified 15 studies, none of which included a radio intervention [3]. A review of 111 mass media campaigns designed to improve maternal and child health in LMICs identified six studies that focused on immunizations, of which only four involved a radio campaign [5]. Importantly, the identified studies, published between 1994 and 2006, showed improved vaccination based on a combination of mass media interventions (i.e., radio and television), interpersonal communication components, and community-based activities [5].

More recently, a high-intensity radio campaign intervention called Saturation+ was implemented and assessed in Burkina Faso, but the study outcomes lacked a focus on infant vaccination [6].

Radio only interventions on vaccination need to be studied in low- and middle-income countries, especially those in sub-Saharan Africa for a number of reasons. First, in many LMICs, radio, unlike television and newspapers, is easily accessible to populations, especially those in rural areas. The 2019/2020 Afrobarometer survey identified radio as the only mass communication channel that bridges the rural-urban divide [7]. The survey found that the percentage of respondents from 18 African countries including Ethiopia who said they listened to radio "every day or a few times a week" was 66 per cent and 67 per cent for rural and urban residents, respectively [7].

In contrast, the survey revealed significant rural versus urban differences in respondent consumption of other media, including television (rural: 35%, urban: 65%), social media (rural: 20%, urban: 48%), the Internet (rural: 18%, urban: 45%), and newspapers (rural: 9%, urban: 22%) [7]. The Afrobarometer survey findings are comparable to other studies, such as the Demographic and Health Survey (DHS) collected from 31 countries in sub-Saharan Africa from 2010 to 2019, which indicated that exposure to mass media channels at least once a week was 57.7% for radio, 39.4% for television and 20.2% for newspapers [8]. Consistent with this differential access, a recent study has found the impact of listening to radio on childhood vaccination in 25 countries in sub-Saharan Africa to be superior to watching television [9].

According to the study, after controlling for several cofounders, respondents who listened to radio at least once a week were 18% more likely to have completed childhood vaccinations compared with those who did not listen to radio, but the corresponding figure for those who watched television at least once a week compared with those who did not watch television at all was only 1%. A second reason that radio-based health information interventions need to be studied further in LMICs is that radio broadcasting is relatively cheap and less limited by literacy as programming can be produced in local languages [10]. Thus, particularly for rural areas which are more likely to include illiterate and low-income individuals, community radio has the potential to be a more tailored and cost-effective method of transmitting health information.

Despite these potential advantages of radio as a health communication medium, to our knowledge, there have been no quasi-experimental trials to test the effect of multipronged radio intervention on childhood vaccination coverage, timeliness, and child health outcomes in a low-income country.

Moreover, the existing literature on the association between behavioural change communication and the coverage of child immunization has given little attention to the impact of community engagement on behavioural change [5]. Thus, there is a need for studies that highlight how community engagement could influence behavioural change—specifically childhood vaccination.

Community engagement interventions tend to involve the priority population in their design and implementation [11]. Thus, radio programmes that require listeners to phone-in have a stronger community engagement appeal than formats such as radio spots that merely disseminate information.

Community engagement has been found to increase child vaccine uptake in LMICs [12]. In Ethiopia, community engagement interventions on childhood vaccination have included community health workers in their delivery [13–15].

A common complaint of behavioural change interventions is the lack of theory that informs their development and implementation [16]. While community health workers have been engaging with parents of infants to aid uptake of childhood vaccinations, and phone-in and drama programmes exist for promoting child health in general, theory-based interventions for promoting childhood vaccination that involve community health workers and radio as a platform for engagement is lacking.

To address this gap, we developed a theory-based "10+10+30" radio intervention for mothers of infants and other community members in Jimma Zone in the Oromia region, Ethiopia that included a 10-minute radio drama on infant vaccination, a 10-minute discussion by community health workers, and a 30-minute phone-in from listeners drama regarding childhood vaccination. The intervention was based on the Health Belief Model (HBM), which emphasizes key constructs that shape health behaviour including perceived benefit, perceived susceptibility, perceived barriers, and cues to action [17, 18]. The HBM suggests that mothers or caregivers who believe that their infants are at risk of vaccine-preventable diseases, and who also believe that their infants would benefit from vaccinations are more likely to adopt the behaviour of vaccinating their infants. The HBM has been commonly applied in studies that assess parental beliefs and attitudes to childhood vaccination [17].

Our goal was to address perceived vaccination barriers and promote infant vaccination coverage and timely administration. The intervention was aired on Jimma Community radio from October 23, 2020 through January 10, 2021. We selected Ethiopia because, despite efforts to achieve full immunization coverage of children, the proportion of fully vaccinated Ethiopian children aged 12–23 months increased from 24% in 2011 to only 39% in 2016 [19]. Factors such as health service use and access to maternal and child health information were found to

predict full immunization coverage [20], thus making a need for communicating with mothers about immunizations particularly critical in this population.

Moreover, the present study was designed to address gaps in communication identified by the Ethiopia Extended Program for Immunization (EPI), including lack of integration with health extension workers and recommended "appropriate strategies of communication, such as Community Conversation, to improve demand for EPI service by the beneficiaries" [20]. Accordingly, this study evaluated the effectiveness of a participatory radio intervention in improving coverage and timeliness of infant vaccination, and reducing morbidity related to vaccine-preventable diseases.

Materials and methods

Ethics statement and trial registration

This study was approved by Ethics Review Board of Jimma University in Ethiopia (IHRPGD/668/2019) and the Institutional Review Board of Texas A&M University (IRB2018-0483D). Women gave oral informed consent to participate in the study. Verbal consent, rather than written consent, was sought because the local research team indicated that it is culturally not appropriate to obtain signatures from most rural households, and that enforcing written informed consent would result in mothers' refusal to participate in the study. Additional information regarding the ethical, cultural, and scientific considerations specific to inclusivity in global research is included in the (S2 File).

In the earlier stages, as this was a quasi-experimental trial involving the use of radio for educating community members on childhood vaccination, the requirement to register it as a clinical trial was not apparent. Thus, despite the endline data collection occurring on January 27–31, 2021, registration of the trial was completed on June 4, 2021. Nevertheless, this late trial registration did not affect the study conduct or results. This trial was registered with ClinicalTrial.gov as NCT04913714.

Intervention development

This prospective, quasi-experimental trial was conducted in two districts (Manna or intervention district and Chora Botor or control district) of Jimma Zone in the Oromia region of Ethiopia. Our intervention, known as "10+10+30", involved 10-minute radio drama on infant vaccination, a 10-minute discussion by community health workers, and a 30-minute phone-in from listeners. The 10+10+30 radio programme on infant vaccination was a radio only multifaceted intervention that aimed to improve vaccination coverage and timeliness.

The 10+10+30's theory of change incorporates two main inter-related causal pathways: a) availability of a local, radio drama serial, with each episode lasting 10 minutes; and b) trained health extension workers (HEWs) to aid a 10-minute discussion of each drama episode on air, and to answer questions from listeners for 30 minutes, thereby improving engagement with priority populations to promote uptake of childhood vaccinations. A trained radio journalist acts as a facilitator for the discussion of the drama, takes questions from listeners, and lets HEWs answer them. In Ethiopia, HEWs work primarily in rural areas, and are the equivalent of community health workers in other countries [21]. A low-level health facility called a health post that serves a population of about 3000 to 5000 in a village (kebele) is assigned two HEWs [22].

The use of education-entertainment contributes to behaviour change and increased uptake of preventive services in sub-Saharan Africa [23]. The '10+10+30' intervention is built upon this principle. It anticipates that if culturally appropriate serial radio drama episodes on vaccination are available, HEWs receive necessary media training to both discuss the drama and to

answer questions from listeners on air, HEWs will reach more mothers or caregivers of infants. In turn, this will encourage mothers/caregivers to vaccinate their infants in a timely manner and promote overall coverage of childhood vaccinations.

The 12 episodes covered content on infant vaccines, vaccine-preventable diseases, and the roles of parents, community leaders, health care professionals and HEWs in promoting timely infant vaccination.

Prior to airing, three of the episodes were pilot tested among 15 mothers (five for each episode) to assess, among other things, their perspectives on cultural appropriateness and duration. During pilot-testing, mothers generally found the drama episodes interesting and educational, and they noted that the 10-minute duration was adequate.

We officially aired “10+10+30” on Jimma Community radio from October 23, 2020 through January 10, 2021. The episodes aired each week on Fridays and Sundays at 2:00 pm. The days and times were chosen based on feedback from the radio drama design workshop. Fridays and Sundays are typically days of rest, and thus husbands or fathers are more likely to be home. Each episode was aired twice; the first time on Fridays, and then again on Sundays as planned. Although the same drama was repeated on both days, the 10-minute discussion and the 30-minute phone-in were unique to each airing. Each 10-minute studio discussion involved two HEWs and a facilitator. Of the ten trained HEWs, two were selected each week to act as radio panellists to discuss the drama, and to answer questions from listeners. Each episode was preceded by a one-minute song with a slogan of “Now more than ever for children”. See S1 File for detailed information on intervention development and implementation.

Study design

To assess the impact of 10+10+30, two districts of Jimma Zone in the Oromia region of Ethiopia were selected as the intervention and control districts. The study districts were strategically selected to ensure that the control (Chora-Botor district) did not have Jimma Community Radio, which was used for the implementation of 10+10+30 radio programme. In other words, Jimma Community Radio’s broadcasting did not reach Chora-Botor district due to the long distance between the district where the radio station was located and the control district [See Fig 1].

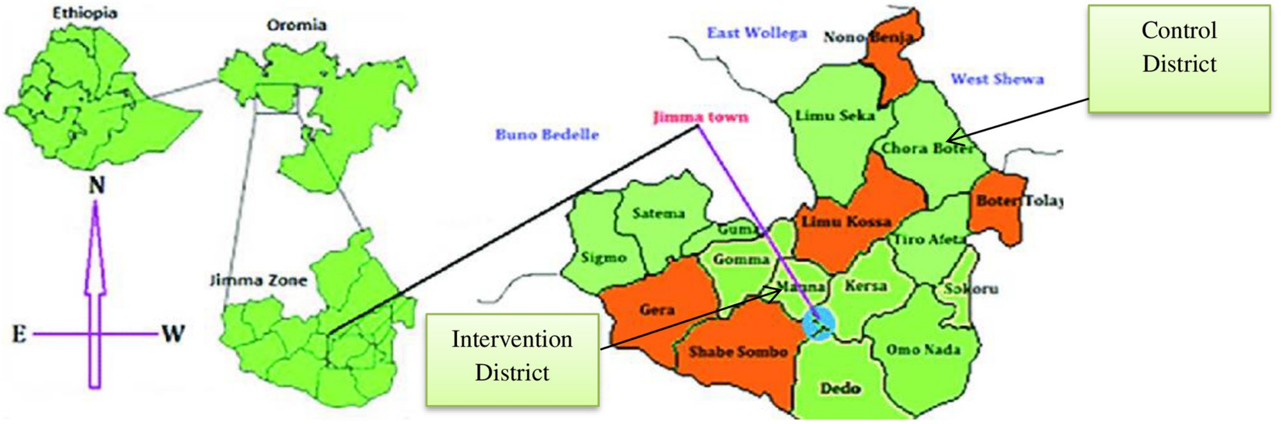

Fig 1. Map of Jimma Zone in Oromia region of Ethiopia showing the intervention and control districts. Source of base map: Kebede Y, Abebe L, Alemayehu G, Sudhakar M, Birhanu (2020) School-based social and behavior change communication (SBCC) advances community exposure to malaria messages, acceptance, and preventive practices in Ethiopia: a pre-posttest study. PLoS One 15(6):e0235189.

https://doi.org/10.1371/journal.pgph.0001002.g001

The episodes were aired on Friday and Sunday at 2:00 pm each week. The days and times were chosen based on the outcome of a formative assessment and the design workshop, which showed that Fridays and Sundays were typical days of rest, and thus husbands—who often owned household radio or mobile phones—were more likely to be at home. The radio drama design workshop also showed that from 2–4 pm, people came together during coffee ceremony, a platform that could be used to listen to the radio [24].

On Jimma Community Radio, sports and entertainment programmes were aired prior to 2 pm. There were no alternative programmes on national radio or other channels which were broadcasting information on child immunization or childcare practices in the two districts. However, there was no information on how often women listen to radio compared to attending community meetings, childcare sessions at health facilities or watching TV in the intervention and control districts.

According to Jimma Zone Health Office, the estimated population of Mana district in 2019 was 202,048 and that of Chora-Botor was 179,077. The number of children under one year old in Mana was estimated to be 6499, with that of Chora-Botor being 5760.

Mothers of infants up to five weeks of age were the main focus of the radio intervention which covered childhood vaccination.

During planning for the intervention, the team agreed to not make access to radio a requirement for recruitment into the study for two reasons. First, the planning phase identified the potential of those listening to the radio programme spreading information to their neighbours. Second, restricting enrolment to those with access to a radio would limit the number of respondents and fail to account for the potential of the project to benefit those who lack access.

A validated questionnaire aimed at collecting information on childhood vaccination and demographic characteristics of households was administered in both districts at baseline (up to 3 days pre-intervention) and 2–3 weeks after the 12 weeks of radio programme were aired (post-intervention). Trained research assistants asked the mothers to complete surveys focused on their child's health, without mentioning the radio campaign. The data collectors were not aware of which participants were in the control or intervention groups to reduce bias.

Both the baseline and post-intervention questionnaires asked respondents whether they had listened to any radio programme in the last 3 months on vaccination that had drama, discussion, and listener phone-in. However, only the post-intervention questionnaire had questions on child health outcomes, including whether in the last 2 weeks their infant had experienced a) diarrhoea, b) fever, c) an illness with a cough, or d) fast, short, rapid breaths or difficulty breathing.

Eligible participants for the survey were mothers ($\geq$18years) who had an infant up to 5 weeks old, lived in the control or intervention districts, and consented orally to be interviewed. Trained data collectors visited households in intervention and control clusters (lowest administrative units or neighbourhoods) to identify all eligible mothers/caregivers (a census) at baseline (October 13–20, 2020) and again at post-intervention (January 27–31, 2021). A census of all eligible mother-infant dyads in the intervention district were visited in their households but two of the kebeles or clusters in the control district were not visited upon reaching the required sample size. The intervention was designed to begin before each child reached 6 weeks old, the recommended age for the first Pentavalent vaccine dose. This vaccine, often referred to as Penta, is a combination of diphtheria, tetanus, whooping cough, hepatitis B and *Haemophilus influenzae* type B vaccines. Penta 1 vaccine is a predictor of subsequent doses in the series (Penta 2 and Penta 3) [25]. Moreover, collecting post-intervention data after the 12-week intervention ensured that the child cohort would be at least 14 weeks old, the recommended age for receiving Penta 3 dose.

Outcomes

The primary outcome was Penta 3 vaccine coverage, which was measured at most 3 weeks after the end of the intervention. Timeliness of the vaccinations was a secondary outcome. Timeliness of vaccination was only assessed among infants with a vaccination date recorded on a health card, and was defined as either early (more than 4 days before the recommended age for vaccination), on-time (from 4 days before the recommended age to 4 weeks after the recommended aged for vaccination), or delayed (more than 4 weeks after the recommended) [26]. The coverage and timeliness of Penta 1, Penta 2, Rotavirus (Rota) 1, Rota 2, Oral Poliovirus (OPV) 1, OPV 2, OPV 3, pneumococcal conjugate vaccine (PCV) 1, PCV 2, and PCV 3 vaccinations were included as secondary outcomes. Any vaccination, either reported on the health card or recalled by the mother, was included as a vaccination. Morbidity indicators of diarrhoea, fever, difficulty breathing, and cough at any time in the prior 2 weeks as reported by the mother were also included as secondary outcomes.

Statistical analysis

The study was powered for the primary outcome: Penta 3 vaccine coverage.

Based on the Penta 3 coverage of 64.0% from both mothers' recall in a survey and use of vaccination cards previously identified in 2017 in Oromia region in Ethiopia [27], we hypothesized that the intervention would lead to a 15% increase in Penta 3 coverage by age 4–5 months. The 15% increase in Penta 3 coverage expected to result from the radio intervention was determined to be significant in Jimma Zone by key health experts during the radio drama design workshop. Our sample size calculations led to a minimum of 337 mother and infant pairs (up to 5 weeks old) per arm (or a total of 674 mother-infant dyads for the two districts), 48 administrative units or clusters per arm (or 96 in total), with at least 7 pairs in each cluster using cluster-randomized sampling.

Our calculations included the following parameters: 1) 80% power, 2) a two-sided alpha of 0.05, 3) an estimated intra- cluster correlation of 0.167, a design effect of 2 relative to simple random sampling, and 10% loss to follow up [28, 29]. An intra-cluster correlation of 0.167 is recommended by the WHO to be a conservative choice for post-campaign vaccine coverage survey if there is no strong reason for selecting another value [28].

Based on population figures in the two districts, rather than random sampling within clusters, we conducted an entire census of the clusters to achieve minimum sample size in the intervention and comparison districts. Also, each district had 22 clusters (hence a total of 44 clusters for the two arms). However, because two of the clusters in the control district were excluded after achieving the sample size for the study, there were 20 clusters in the control district and 22 clusters in the intervention district (Fig 2).

Two analyses were completed: an intention-to-treat as the primary analysis and a per-protocol as the secondary analysis. In the intention-to-treat analysis, all mothers in the intervention district were considered exposed to the radio program and all mothers in the control district were considered not exposed to the radio program. In the per-protocol analysis, mothers in the intervention district who reported listening to any radio programme on vaccination that had drama, discussion and listener phone-in during the past three months on the post-intervention survey were considered exposed to the radio programme. Mothers in the control district who did not report listening to any radio programme on vaccination that had drama, discussion and listener phone-in during the past three months on the post-intervention survey were considered not exposed to the radio program. All other mother-infant dyads were eliminated from the per-protocol analysis.

We compared characteristics of the two groups for factors that have been shown to be associated with increased rates of vaccination from the literature in low- and middle-income

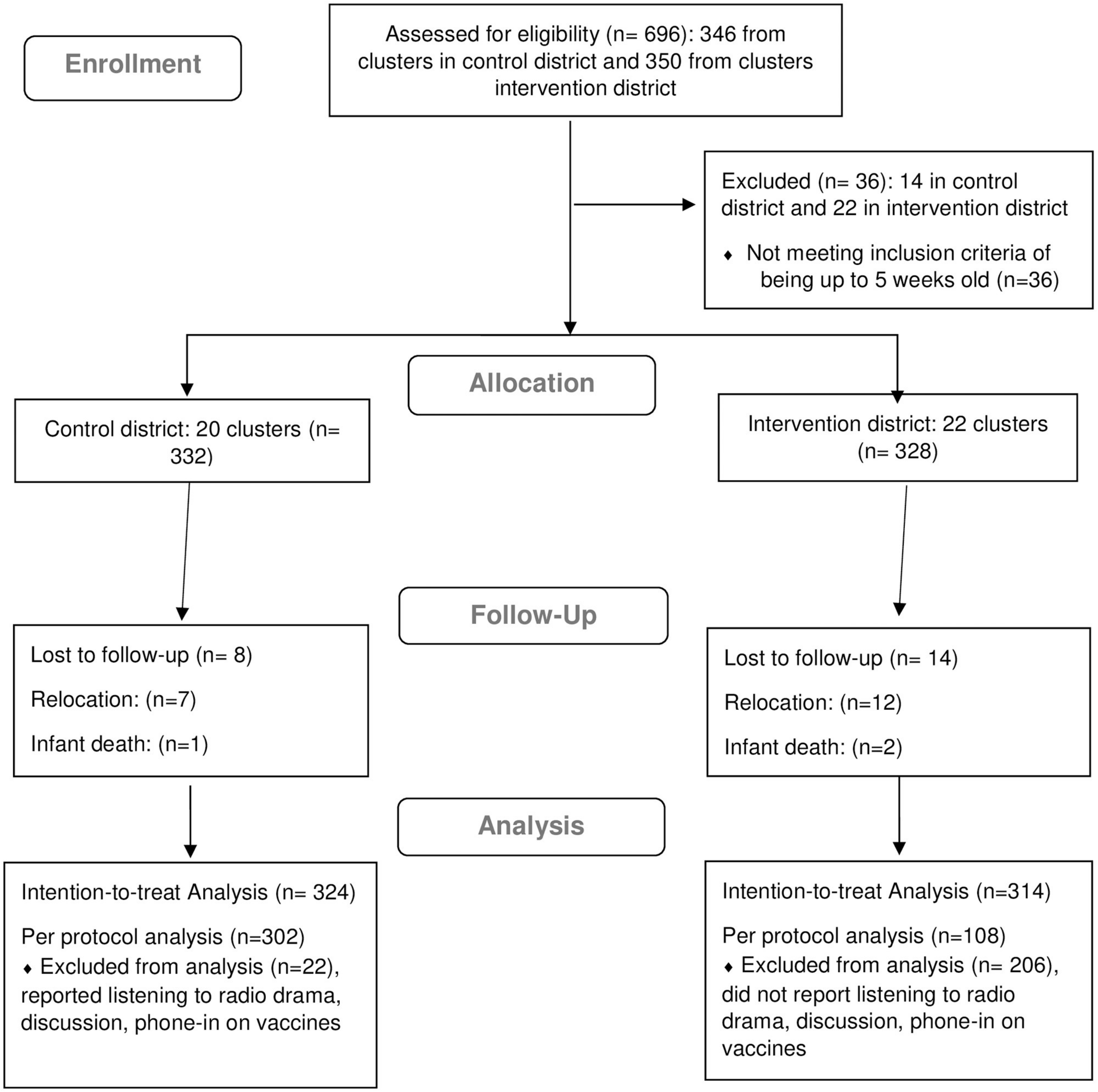

Fig 2. Flow diagram of 10+10+30 intervention evaluation.

https://doi.org/10.1371/journal.pgph.0001002.g002

countries [30], and Ethiopia in particular [31–33]. These factors included sex of child (male/female), birth order (first, second or third, and fourth or later), religion (Islam, Orthodox, other), marital status (yes/no), location (urban/rural), walking time to vaccination centre (<15 minutes, 15–30 minutes, >30 minutes), place of delivery (home/health centre), frequency of antenatal care (0 visits, 1–3 visits, >3 visits), vaccination verification card (yes/no), age of child at baseline (in days), age of mother at baseline (in years), wealth index (number of yes responses to 18 questions about household commodities, livestock, and land), and frequency of radio listenership. These factors were chosen a-priori [34].

A sensitivity analysis was completed where only children with vaccinations with dates that were recorded on the health card were considered to be vaccinated. All others, including those where the health card indicated a vaccination, but no date was recorded and those where the mother recalled the vaccination, were considered to not be vaccinated for the purpose of the sensitivity analysis.

Categorical variables were compared using a $\chi2$ test and continuous variables compared using a t-test. A generalized linear model with a log link and Poison distribution with robust variances was used to compare the risk of the various outcomes between the intervention and control arms. We expect that our outcomes are likely to be correlated within kebeles (lowest administrative units) or neighbourhoods. Therefore, the lowest administrative units were included in the regression models as a random intercept to account for this clustering (correlation) within neighbourhoods. Regression models were both adjusted and unadjusted for the confounders described above. Anyone with missing data or a response of "don't know" was eliminated from the regression that included the variable of interest. The same analysis was repeated for both the intention to treat analysis, the per-protocol analysis, and the sensitivity analysis.

Timeliness of vaccination was only examined in cases where the date of vaccination was recorded on the health card. Two-sided p-values less than 0.05 were considered statistically significant. All analysis was completed using Stata version 15 [35].

Results

Overall, 696 women were assessed for eligibility, and 332 women-infant dyads in the control district and 328 women-infant dyads from the intervention district were enrolled at baseline (Fig 2). Although we estimated 7 infants per cluster, in practice the number of infants per cluster varied, in some cases being nearly three times as large.

At post-intervention, data were completed for 324 (97.6%) and 314 (95.7%) women-infant dyads across 20 lowest administrative units in the control and 22 lowest administrative in the intervention district, respectively. Of these, 302/324 (93.2%) of the control dyads and 108/314 (34.4%) of the intervention dyads were included in the per-protocol analysis based on self-reported exposure to the intervention. The control and intervention groups had some significant differences across demographic and confounding factors (Table 1). Notably, the intervention group had higher educational attainment, was more likely to live in an urban area, was closer to the vaccination site, was more likely to have delivered in a health facility, and had greater numbers of antenatal care visits. Similar trends were observed in the per protocol analysis, although the groups tended to be more balanced across the demographic and confounding factors (S1 Table).

Childhood vaccine coverage

As shown in Table 2, according to the intent-to-treat analysis Penta 3 vaccine coverage was significantly higher in the intervention group (89.8%, 95% CI: 85.9–92.9%) compared with the control group (65.1%, 95% CI: 59.7–70.3%). Similar results were obtained in the per-protocol analysis, with the intervention group showing coverage of 97.2% (95% CI: 92.1–99.4%) versus 62.9% (95% CI: 57.2–68.4%). Indeed, vaccination rates were significantly higher across all of the childhood vaccinations examined in both the intention-to-treat and per-protocol analyses. In a sensitivity analysis, where only children with vaccination dates recorded on a health card were considered, the proportion vaccinated was lower, but similar trends were observed (S2 Table).

As shown in Table 3, in the intention-to-treat regression analysis, infants were 35% more likely to have received Penta 3 vaccination in the intervention group than in the control group (adjusted RR: 1.35, 95% CI: 1.21–1.50). A similar pattern of significant effects was observed

Table 1. Baseline characteristics of the participants in the intention to treat analysis.

CHARACTERISTICS	CONTROL (N (%)) N = 324	INTERVENTION (N (%)) N = 314	X^2 P-VALUE
Sex of the child			0.68
Boy	167 (51.5%)	167 (53.2%)	
Girl	157 (48.5%)	147 (46.8%)	
Birth order			0.54
1st	83 (25.6%)	83 (26.4%)	
2nd-3rd	132 (40.7%)	138 (43.9%)	
4+	109 (33.6%)	93 (29.6%)	
Education			<0.001
No formal education	156 (48.8%)	102 (32.5%)	
Primary	102 (31.9%)	130 (41.4%)	
Secondary or higher	62 (19.4%)	82 (26.1%)	
Religion			<0.001
Islam	230 (71.0%)	284 (90.4%)	
Orthodox	59 (18.2%)	18 (5.7%)	
Other	35 (10.8%)	12 (3.8%)	
Marital Status			0.093
Married	320 (98.8%)	304 (96.8%)	
Other	4 (1.2%)	10 (3.2%)	
Residence			0.010
Rural	295 (91.0%)	265 (84.4%)	
Urban	29 (9.0%)	49 (15.6%)	
Time to vaccination site			<0.001
< 15 Minutes	61 (18.8%)	104 (33.1%)	
15–30 Minutes	146 (45.1%)	139 (44.3%)	
> 30 Minutes	117 (36.1%)	71 (22.6%)	
Place of Birth			0.006
Yours, relative's, or neighbour's home	87 (26.9%)	56 (17.8%)	
Hospital, Health Center, or Health Post	237 (73.1%)	258 (82.2%)	
Frequency of ANC			<0.001
No	44 (13.6%)	14 (4.5%)	
1–3	169 (52.2%)	158 (50.3%)	
4+	111 (34.3%)	142 (45.2%)	
Card Verified			<0.001
Verified	232 (71.6%)	114 (36.3%)	
Not Verified	92 (28.4%)	200 (63.7%)	
Household Radio Ownership	141 (43.5%)	173 (55.1%)	0.003
Frequency of Listening to the Radio			<0.001
Almost every day	13 (4.0%)	46 (14.6%)	
At least once a week	18 (5.6%)	69 (22.0%)	
Less than once a week	21 (6.5%)	38 (12.1%)	
Not at all	272 (84.0%)	161 (51.3%)	
Household Mobile Phone Ownership	142 (43.8%)	233 (74.2%)	<0.001
	**MEAN (SD)**	**MEAN (SD)**	**T-TEST P-VALUE**
Age of the Child (days)	16.59 (10.58)	18.85 (12.08)	0.012
Age of mother (years)	26.73 (6.11)	25.91 (5.09)	0.067
Wealth Index	6.36 (2.13)	7.28 (2.89)	<0.001

https://doi.org/10.1371/journal.pgph.0001002.t001

Table 2. Vaccination outcomes in the control and intervention groups.

	INTENTION-TO-TREAT ANALYSES			PER-PROTOCOL ANALYSES		
	Control n = 324	Intervention n = 314	X^2	Control n = 302	Intervention n = 108	X^2
Vaccine	N (%)	N (%)	P-VALUE	N (%)	N (%)	P-VALUE
Penta3	211 (65.1%)	282 (89.8%)	<0.001	190 (62.9%)	105 (97.2%)	<0.001
Penta2	219 (66.6%)	293 (93.3%)	<0.001	198 (65.6%)	105 (97.2%)	<0.001
Penta1	219 (67.6%)	293 (93.3%)	<0.001	207 (68.5%)	105 (97.2%)	<0.001
Rota2	217 (67.0%)	293 (93.3%)	<0.001	196 (64.9%)	105 (97.2%)	<0.001
Rota1	223 (68.8%)	300 (95.5%)	<0.001	202 (66.9%)	105 (97.2%)	<0.001
PCV3	95 (29.3%)	225 (71.7%)	<0.001	93 (30.8%)	85 (78.7%)	<0.001
PCV2	217 (67.0%)	293 (93.3%)	<0.001	196 (64.9%)	105 (97.2%)	<0.001
PCV1	226 (69.8%)	300 (95.5%)	<0.001	205 (67.9%)	105 (97.2%)	<0.001
OPV3	209 (64.5%)	289 (92.0%)	<0.001	188 (62.3%)	108 (100.0%)	<0.001
OPV2	209 (64.5%)	295 (93.9%)	<0.001	188 (62.3%)	108 (100.0%)	<0.001
OPV1	224 (69.1%)	303 (96.5%)	<0.001	203 (67.2%)	108 (100.0%)	<0.001
Fully Vaccinated[a]	95 (29.3%)	223 (71.0%)	<0.001	93 (30.8%)	85 (78.7%)	<0.001

[a]Fully vaccinated was defined as receiving all doses of Penta, Rota, PCV, and OPV (excluding birth dose).

https://doi.org/10.1371/journal.pgph.0001002.t002

across all of the vaccines examined. Table 3 also shows that, in the per-protocol regressions, the risk of Penta 3 vaccination was 40% higher in the intervention versus control group (adjusted RR: 1.40, 95% CI: 1.33–1.68), and a similar pattern was once again observed across all types of vaccination assessed.

In the sensitivity analysis that were restricted to children with vaccination dates recorded on a health card, the likelihood of completing Penta 3 vaccination was 1.96 times higher in the intervention compared to the control (adjusted RR: 1.96, 95% CI: 1.57–2.45) (S3 Table). The likelihood of completing Penta 3 vaccination was higher in the sensitivity analysis as compared to the main analysis, but the risks of other vaccinations were generally lower in the sensitivity analysis. The intervention was associated with significantly higher likelihood of completing all vaccinations examined in both the intention to treat and per-protocol cohorts in the sensitivity analysis (S3 Table).

Childhood vaccine timeliness

As shown in Table 4, the intention-to-treat analyses revealed that the intervention was also associated with enhanced Penta 3 vaccination timeliness. Specifically, among infants with recorded vaccination dates, the proportion of infants receiving their Penta 3 vaccine on time was 60.1% in the intervention group (95% CI: 52.6–67.2%) versus 47.8% (95% CI: 37.7–58.1%) in the control group. Most of the other infant vaccines, with the exception of OPV3 which was not statistically significant, were also had better timeliness in the intervention group as compared to the control group. Finally, Table 4 also shows that similar results were obtained for the per-protocol analyses.

Infant morbidity

As shown in Table 5, infants in the intervention group had fewer reported cases of illness in the previous two weeks across all symptoms examined according to both the intention-to-treat and per-protocol cohorts.

Compared with infants in the control group, intention to treat analysis after adjusting for sex of child, birth order, religion, marital status, urban/rural, time to vaccination centre, place

Table 3. Regression analysis for vaccination outcomes comparing the intervention to control groups.

	INTENTION TO TREAT (N = 638)						PER PROTOCOL (N = 410)					
OUTCOME	UN-ADJUSTED RR	95% CI	P-VALUE	ADJUSTED RR[B]	95% CI	P-VALUE	UN-ADJUSTED RR	95% CI	P-VALUE	ADJUSTED RR[B]	95% CI	P-VALUE
Penta 3	1.39	1.26–1.52	<0.000	1.35	1.21–1.50	<0.000	1.56	1.41–1.72	<0.000	1.40	1.33–1.68	<0.000
Penta 2	1.40	1.27–1.53	<0.000	1.35	1.21–1.51	<0.000	1.50	1.34–1.65	<0.000	1.46	1.30–1.64	<0.000
Penta 1	1.37	1.26–1.50	<0.000	1.33	1.21–1.47	<0.000	1.43	1.30–1.58	<0.000	1.38	1.23–1.54	<0.000
Rota 2	1.41	1.28–1.55	<0.000	1.36	1.22–1.52	<0.000	1.52	1.37–1.68	<0.000	1.48	1.31–1.66	<0.000
Rota 1	1.41	1.28–1.54	<0.000	1.37	1.24–1.52	<0.000	1.47	1.33–1.63	<0.000	1.42	1.27–1.58	<0.000
PCV 3	2.46	2.06–2.93	<0.000	2.51	2.09–3.04	<0.000	2.54	2.07–3.13	<0.000	2.86	2.36–3.48	<0.000
PCV 2	1.41	1.28–1.54	<0.000	1.36	1.22–1.52	<0.000	1.51	1.37–1.67	<0.000	1.47	1.31–1.66	<0.000
PCV 1	1.38	1.27–1.51	<0.000	1.34	1.22–1.49	<0.000	1.45	1.32–1.60	<0.000	1.39	1.25–1.54	<0.000
OPV 3	1.44	1.31–1.58	<0.000	1.39	1.25–1.56	<0.000	1.63	1.47–1.79	<0.000	1.56	1.39–1.74	<0.000
OPV 2	1.47	1.34–1.62	<0.000	1.44	1.28–1.61	<0.000	1.63	1.47–1.79	<0.000	1.57	1.40–1.76	<0.000
OPV 1	1.40	1.29–1.53	<0.000	1.39	1.26–1.53	<0.000	1.49	1..37–1.64	<0.000	1.47	1.32–1.63	<0.000
Fully Vaccinated[a]	2.43	2.04–2.90	<0.000	2.49	2.07–3.01	<0.000	2.54	2.07–3.13	<0.000	2.86	2.36–3.48	<0.000

[a] Fully vaccinated was defined as receiving all doses of Penta, Rota, PCV, and OPV (excluding birth dose).

[b]Adjusted for sex of child, birth order, religion, marital status, urban/rural, time to vaccination centre, place of delivery, antenatal care, able to see age/vaccination verification card, age of child in days at baseline, age of mother in years, radio listening, wealth index, and accounting for clustering within kebeles. Unadjusted models still accounted for clustering within kebeles.

RR: Risk Ratio

CI: Confidence Interval

https://doi.org/10.1371/journal.pgph.0001002.t003

of delivery, antenatal care, able to see age/vaccination verification card, age of child in days at baseline, age of mother in years, radio listening, wealth index, and clustering within kebeles showed that children in the intervention group were significantly 75% less likely to experience diarrhoea (RR: 0.25, 95% CI: 0.15–0.42), 42% less likely to experience fever (RR: 0.58, 95% CI: 0.44–0.76), 55% less likely to have a cough (RR: 0.45, 95% CI: 0.35–0.56), and 44% less like to have difficulty breathing (RR: 0.56, 95% CI: 0.43–0.73). The intervention group was also less likely to experience symptoms than the control group in the per-protocol cohort, although the magnitude of the association tended to be larger than in the intention-to-treat analysis. In per protocol, children in the intervention group were significantly 60% less likely to experience cough (RR: 0.40, 95% CI: 0.25–0.64), 50% less likely to have a fever (RR: 0.50, 95% CI: 0.29–0.86), and 58% less like to have difficulty breathing (RR: 0.42, 95% CI: 0.23–0.77).

Discussion

The present study provides promising evidence that a "10+10+30" radio intervention is associated with increased infant vaccine coverage and timeliness, and may help reduce morbidity.

Table 4. Timeliness of infant vaccinations for control and intervention groups among those with a recorded vaccination date.

	INTENTION TO TREAT							PER PROTOCOL						
	CONTROL			INTERVENTION				CONTROL			INTERVENTION			
VACCINE	ON-TIME N (%)	EARLY N (%)	LATE N (%)	ON-TIME N (%)	EARLY N (%)	LATE N (%)	X^2 P-VALUE	ON-TIME N (%)	EARLY N (%)	LATE N (%)	ON-TIME N (%)	EARLY (N (%))	LATE N (%)	X^2 P-VALUE
Penta3	44 (47.8%)	48 (52.2%)	0 (0.0%)	104 (60.1%)	47 (27.2%)	22 (12.7%)	<0.001	42 (47%)	48 (53%)	0 (0%)	50 (66%)	14 (18%)	12 (16%)	<0.001
Penta2	94 (55.0%)	50 (29.2%)	27 (15.8%)	161 (77.8%)	31 (15.0%)	15 (7.2%)	<0.001	82 (54.3%)	48 (31.8%)	21 (13.9%)	77 (87.5%)	5 (5.7%)	6 (6.8%)	<0.001
Penta1	82 (43.9%)	93 (49.7%)	12 (6.4%)	172 (72.0%)	57 (23.8%)	10 (4.2%)	<0.001	63 (38.0%)	91 (54.8%)	12 (7.2%)	84 (89.4%)	10 (10.6%)	0 (0.0%)	<0.001
Rota2	94 (54.3%)	50 (28.9%)	29 (16.8%)	161 (77.4%)	31 (14.9%)	16 (7.7%)	<0.001	82 (53.6%)	48 (31.4%)	23 (15.0%)	77 (87.5%)	5 (5.7%)	6 (6.8%)	<0.001
Rota1	82 (44.8%)	89 (48.6%)	12 (6.6%)	172 (72.3%)	56 (23.5%)	10 (4.2%)	<0.001	63 (38.9%)	87 (53.7%)	12 (7.4%)	84 (89.4%)	10 (10.6%)	0 (0.0%)	<0.001
PCV3	45 (49.5%)	46 (50.5%)	0 (0.0%)	103 (60.2%)	46 (26.9%)	22 (12.9%)	<0.001	43 (48%)	46 (52%)	0 (0%)	48 (65%)	14 (19%)	12 (16%)	<0.001
PCV2	92 (55.1%)	48 (28.7%)	27 (16.2%)	160 (77.7%)	31 (15.0%)	15 (7.3%)	<0.001	80 (54.4%)	46 (31.3%)	21 (14.3%)	77 (87.5%)	5 (5.7%)	6 (6.8%)	<0.001
PCV1	82 (44.8%)	89 (48.6%)	12 (6.6%)	172 (72.0%)	57 (23.8%)	10 (4.2%)	<0.001	63 (38.9%)	87 (53.7%)	12 (7.4%)	84 (89.4%)	10 (10.6%)	0 (0.0%)	<0.001
OPV3	15 (57.7%)	11 (42.3%)	0 (0.0%)	113 (59.2%)	53 (27.7%)	25 (13.1%)	0.08	15 (58%)	11 (42%)	0 (0%)	51 (60%)	22 (26%)	12 (14%)	0.06
OPV2	71 (54.2%)	45 (34.4%)	15 (11.5%)	185 (74.0%)	47 (18.8%)	18 (7.2%)	<0.001	59 (53.2%)	43 (38.7%)	9 (8.1%)	42 (41.2%)	55 (53.9%)	5 (4.9%)	<0.001
OPV1	61 (49.6%)	57 (46.3%)	5 (4.1%)	186 (69.9%)	68 (25.6%)	12 (4.5%)	<0.001	80 (80.0%)	13 (13.0%)	7 (7.0%)	87 (82.1%)	18 (17.0%)	1 (0.9%)	<0.001

https://doi.org/10.1371/journal.pgph.0001002.t004

Table 5. Childhood illness outcomes in the control and intervention groups.

	INTENTION TO TREAT			PER PROTOCOL		
	CONTROL	INTERVENTION	X^2 P-VALUE	CONTROL	INTERVENTION	X^2 P-VALUE
Diarrhoea (N (%))	111 (35.7%)	19 (6.6%)	<0.001	111 (38.3%)	4 (4.1%)	<0.001
N Missing	13	27		12	10	
Fever (N (%))	84 (26.0%)	57 (18.2%)	0.02	81 (26.9%)	18 (16.7%)	0.02
N Missing	1	0		1	0	
Cough (N (%))	110 (34.1%)	54 (17.2%)	<0.001	109 (36.2%)	18 (16.7%)	<0.001
N Missing	1	0		1	0	
Difficulty breathing (N (%))	94 (29.2%)	55 (17.6%)	<0.001	91 (30.3%)	15 (13.9%)	<0.001
N Missing	2	1		2	0	

https://doi.org/10.1371/journal.pgph.0001002.t005

The positive effect on vaccine coverage reflects similar results obtained for mass media campaigns in low- and middle-income countries such as Bangladesh, Ecuador, Peru, Philippines and the Democratic Republic of Congo [5]. However, unlike previous studies that used combinations of mass media, and thus making the effect of the radio component alone difficult to establish, the current used only radio, making it possible to quantify potential of radio only intervention in improving childhood vaccination coverage. For example, Hutchinson and colleagues assessed the effect of 26 episodes of a television serial drama with radio spots, newspapers, billboards, community-level activity promotions in rural Bangladesh from Aug 2001 to March 2002 [36]. Results indicated that DPT3 vaccine coverage among those exposed to the interventions was 64% versus 48% among those not exposed. In the current study, intention-to-treat analysis indicated that the coverage of Penta 3 vaccine (which contains two more antigens than a third dose of DPT3 vaccine) was 89.8% among participants in a region exposed to the intervention versus 65.1% among participants in a control region without access to the intervention. Combined with similar positive findings for the per-protocol analysis, the current findings provide initial empirical support for the 10+10+30 radio intervention as a method of significantly increasing infant vaccine coverage.

Timely completion of childhood vaccination is also a key goal in averting vaccine-preventable diseases. Penta 3 vaccine is often a primary focus of childhood vaccination because its completion indicates coverage of the first three series of the childhood vaccinations at ages 6 weeks, 10 weeks and 14 weeks. In the current study, intention-to-treat analysis found that on-time completion of Penta 3 vaccine was 60.1% among participants in the intervention district and 47.8% in the control district, with per-protocol analysis having coverages of 66% and 47%, respectively. To our knowledge, this is the first experimental trial to assess timeliness of Penta 3 vaccines with a radio-only intervention in a low-income country. Our results suggest that listeners exposed to the programming about the benefits of vaccinating their children and the dangers of not doing so were encouraged to have their infants vaccinated in a timely manner. This finding is consistent with results from a recent study of the effect of mass media exposure on childhood immunization coverage in low- and middle-income countries using pooled cross-sectional data from the Demographic Health Surveys of 13 countries in sub-Saharan Africa for the years 2004–2010 [4].

Systematic reviews of interventions for improving infant vaccination had not identified a single study that focused only on radio [3, 5], making our study highly innovative in its design and outcomes. In a separate study in Pakistan that did not use radio but had similar design and outcomes like those of the current study, exposure to pictorial cards to increase the

knowledge of mothers about vaccination was significantly associated with improved infant vaccination compared with the control [37]. In that study, researchers followed mother-infant dyads from less than 6 weeks of age until 4 months, with 72.1% in the intervention group completing all 3 doses of DPT/Hepatitis B vaccine as compared with only 51.7% in the control group [37].

The current study's focus on the relationship between exposure to radio-only intervention on vaccination and child morbidity outcomes is also novel, but shares attributes of a child health radio campaign called Saturation+ which was implemented in Burkina Faso. Although Saturation+ did not focus on vaccination, it did focus on similar child morbidity outcomes including fever, diarrhoea, and fast or difficult breathing within two weeks prior to data collection [6]. Relative to the present study, Saturation+ was a more resource intense approach that included airing 1-minute spots at least 10 times per day and a 2-hour programme airing 5 nights per week that involved a 10-minute live radio drama acted on location by local actors and remainder of the programme taken up by news, music, and discussion [6, 38]. Although mothers in the Saturation+ intervention group reported fewer episodes of fever (58.2% versus 73.5%), diarrhoea (54.1% versus 64.4%), and fast/difficult breathing (48.1% versus 54.1%) in their children relative to control mothers, none of the differences was statistically significant [6]. Thus, it is particularly noteworthy that the present '10+10+30' intervention, which was less resource intensive, may be associated with reduced child morbidity. However, the cost-effectiveness of 10+10+30 has not yet been determined unlike Saturation+ [39].

The '10+10+30' intervention campaign seems to have reached a third of the primary target population given that 108 of the 314 mothers (34.4%) in the intervention district reported having listened to a radio programme with drama, discussion and phone-in segments on childhood vaccination. However, 22 of the 324 mothers (6.8%) in the control district also reported having listened to the '10+10+30' intervention even though the control district did not have such a programme. This may suggest possible confusion with other radio programmes, a tendency to provide socially-desirable answers, or perhaps a lack of understanding of the question. Regardless of the source, the per-protocol analyses excluded those in the control district who reported listening to the programme and included only those in the intervention district who reported that they listened the programme. Hence, the continuing presence of significant effects when this potential source of variability is accounted for provides further support for the benefit among those who actually received the intervention.

Limitations

Although the '10+10+30' radio intervention appears to have promise as a strategy to enhance infant vaccination coverage, timeliness and related morbidity, our findings should be interpreted with caution because of some limitations. First and foremost, despite carefully selecting the control and intervention districts, there were important differences in some key sociodemographic factors that are known to affect childhood vaccination. Although we statistically controlled for these differences in our analyses, statistical control does not guarantee the generalizability of the findings to other communities. Second, due to the low number of infants in both control and intervention districts, a census of the clusters and households were used rather than randomization. Given that two of the kebeles or clusters in the control district were not visited upon reaching the required sample size, selection bias could have affected the results of the study. Third, there were relatively few people with missing data, and thus such observations were eliminated. Using such an approach for handling missing data may have implications for the power of the study [40]. Fourth, the lack of information on how often women in the control and intervention districts listen to radio compared to attending

community meetings, childcare sessions at health facilities or watching TV may also lead to confounding errors in the two groups.

Fifth, we relied on parental recall of childhood vaccination status in instances when there was no vaccination card available. While this is a common procedure in some childhood vaccination studies, it nonetheless presents a challenge related to recall bias [30]. Unfortunately, requiring a vaccination card as confirmatory evidence may not be feasible in some populations, and particularly in rural Ethiopia [41]. For example, the Ethiopian Demographic Health Survey of 2016 found that only 25.9% of children aged 12–23 months in the Oromia region had vaccination cards during home visit [42]. Lastly, our reliance of maternal recall of infant illness symptoms during a preceding the two-week period may also be subject to reporting errors; however, efforts to gauge morbidity using other metrics (e.g., medical visits) would introduce other potential errors such as omission of milder symptoms and individual differences in access to, or threshold for seeking, medical care.

Conclusions

In summary, the current study provides initial evidence that the '10+10+30' radio intervention is associated with increased infant vaccination coverage, timeliness and reduced morbidity. Whereas caution is needed in interpreting the present findings, most importantly because of demographic differences between the intervention and control group, this study nonetheless contributes to the literature by providing the first evidence that a radio-only intervention (10+10+30) may increase infant vaccinations in a low-income country. Additional randomised controlled trials are needed to verify and extend the present results and to examine population-level changes.

Supporting information

S1 Table. Baseline characteristics of the participants in the per protocol analysis.
(DOCX)

S2 Table. Vaccination outcomes in the control and intervention groups.
(DOCX)

S3 Table. Regression analysis for vaccination outcomes comparing the intervention to control groups.
(DOCX)

S1 File. Development and implementation of 10+10+30 radio campaign on child vaccination in Ethiopia.
(DOCX)

S2 File. Inclusivity questionnaire.
(DOCX)

Acknowledgments

We wish to thank all the participants who participated in radio drama design workshop and contributed to the discussions that led to creation of the scripts. In particular, we wish to thank Mrs. Seralem Genet, and Mr. Getnet Bayih of the World Health Organization (WHO)-Ethiopia Office for making inputs into the creation of radio drama episodes.

Author Contributions

Conceptualization: Bernard Appiah.

Formal analysis: Abebe Mamo, Brittany Kmush.

Funding acquisition: Bernard Appiah.

Investigation: Bernard Appiah, Lakew Abebe Gebretsadik, Elfreda Samman, Tena Alemayehu, David Larsen.

Methodology: Bernard Appiah, Lakew Abebe Gebretsadik, Abebe Mamo, Yisalemush Asefa, Laura Forastiere.

Project administration: Bernard Appiah, Lakew Abebe Gebretsadik, Yisalemush Asefa, Mahdiya Abafogi.

Supervision: Lakew Abebe Gebretsadik, Abebe Mamo.

Validation: Abebe Mamo, Brittany Kmush.

Writing – original draft: Bernard Appiah, Lakew Abebe Gebretsadik, Brittany Kmush, Christopher R. France, Md Koushik Ahmed, Gursimar Kaur Singh.

Writing – review & editing: Bernard Appiah, Lakew Abebe Gebretsadik, Abebe Mamo, Brittany Kmush, Yisalemush Asefa, Christopher R. France, Elfreda Samman, Tena Alemayehu, Mahdiya Abafogi, Md Koushik Ahmed, Laura Forastiere, Gursimar Kaur Singh, David Larsen.

References

1. Ozawa S, Clark S, Portnoy A, Grewal S, Brenzel L, Walker DG. Return on investment from childhood immunization in low-and middle-income countries, 2011–20. Health Aff. 2016; 35(2):199–207. https://doi.org/10.1377/hlthaff.2015.1086 PMID: 26858370
2. Gandhi G, Lydon P, Cornejo S, Brenzel L, Wrobel S, Chang H. Projections of costs, financing, and additional resource requirements for low-and lower middle-income country immunization programs over the decade, 2011–2020. Vaccine. 2013; 31:B137–48. https://doi.org/10.1016/j.vaccine.2013.01.036 PMID: 23598475
3. Oyo-Ita A, Wiysonge CS, Oringanje C, Nwachukwu CE, Oduwole O, Meremikwu MM. Interventions for improving coverage of childhood immunisation in low-and middle-income countries. Cochrane Database Syst Rev. 2016;(7). https://doi.org/10.1002/14651858.CD008145.pub3 PMID: 27394698
4. Jung M, Lin L, Viswanath K. Effect of media use on mothers' vaccination of their children in sub-Saharan Africa. Vaccine. 2015; 33(22):2551–7. https://doi.org/10.1016/j.vaccine.2015.04.021 PMID: 25896379
5. Naugle DA, Hornik RC. Systematic review of the effectiveness of mass media interventions for child survival in low-and middle-income countries. J Health Commun. 2014; 19(sup1):190–215. https://doi.org/10.1080/10810730.2014.918217 PMID: 25207453
6. Sarrassat S, Meda N, Badolo H, Ouedraogo M, Some H, Bambara R, et al. Effect of a mass radio campaign on family behaviours and child survival in Burkina Faso: a repeated cross-sectional, cluster-randomised trial. Lancet Glob Heal. 2018; 6(3):e330–41. https://doi.org/10.1016/S2214-109X(18)30004-4 PMID: 29433668
7. Conroy-Krutz J, Koné J. Promise and peril: In changing media landscape, Africans are concerned about social media but opposed to restricting access. 2020 [Cited 14 July 2022]. https://www.afrobarometer.org/wp-content/uploads/migrated/files/publications/Dispatches/ad410-promise_and_peril-africas_changing_media_landscape-afrobarometer_dispatch-2dec20.pdf
8. Mutumba M. Mass media influences on family planning knowledge, attitudes and method choice among sexually active men in sub-Saharan Africa. PLOS ONE. 2022 Jan 27; 17(1):e0261068. https://doi.org/10.1371/journal.pone.0261068 PMID: 35085245
9. Bobo FT, Asante A, Woldie M, Dawson A, Hayen A. Child vaccination in sub-Saharan Africa: Increasing coverage addresses inequalities. Vaccine. 2022 Jan 3; 40(1):141–50. https://doi.org/10.1016/j.vaccine.2021.11.005 PMID: 34794824

10. Myers M. Voices from villages: Community radio in the developing world. Center for International Media Assistance. 2011 Apr 5.
11. Questa K, Das M, King R, Everitt M, Rassi C, Cartwright C, et al. Community engagement interventions for communicable disease control in low-and lower-middle-income countries: evidence from a review of systematic reviews. Int. J. Equity Health. 2020; 19(1):1–20. https://doi.org/10.1186/s12939-020-01169-5 PMID: 32252778
12. Jain M, Shisler S, Lane C, Bagai A, Brown E, Engelbert M, et al. Use of community engagement interventions to improve child immunisation in low-and middle-income countries: A systematic review and meta-analysis. Campbell Systematic Reviews. 2022; 18(3):e1253.
13. Demissie SD, Kozuki N, Olorunsaiye CZ, Gebrekirstos P, Mohammed S, Kiapi L, et al. Community engagement strategy for increased uptake of routine immunization and select perinatal services in north-west Ethiopia: A descriptive analysis. PLOS ONE. 2020 29; 15(10):e0237319. https://doi.org/10.1371/journal.pone.0237319 PMID: 33119604
14. Bayih G, Teklu A, Mekonnen ZA, Tsedaw T, Tefera S, Feletto M, et al. The Implementation of Social and Behavior Change Communication Intervention to Improve Immunization Demand: A qualitative study in Awabel District, Northwest Ethiopia. Ethiopian Journal of Health Development. 2021; 35(3).
15. Kerebih M, Minyihun A, Gudale A, Shiferaw A, Hagos A, Mekonnen ZA, et al. How to optimize health facilities and community linkage in order to enhance immunization service? The case of West Amhara Region, Ethiopia. Ethiopian Journal of Health Development. 2021; 35(3).
16. Glanz K, Bishop DB. The role of behavioral science theory in development and implementation of public health interventions. Annual review of public health. 2010; 31(1):399–418. https://doi.org/10.1146/annurev.publhealth.012809.103604 PMID: 20070207
17. Dyda A, King C, Dey A, Leask J, Dunn AG. A systematic review of studies that measure parental vaccine attitudes and beliefs in childhood vaccination. BMC Public Health. 2020; 20(1):1–8.
18. Vermandere H, van Stam M-A, Naanyu V, Michielsen K, Degomme O, Oort F. Uptake of the human papillomavirus vaccine in Kenya: testing the health belief model through pathway modeling on cohort data. Global Health. 2016; 12(1):1–13. https://doi.org/10.1186/s12992-016-0211-7 PMID: 27846857
19. Federal Ministry of Health. Ethiopia national expanded programme on immunization: Comprehensive multi- comprehensive multi-year plan 2016 year plan 2016 year plan 2016–2020. Addis Ababa: Federal Ministry of Health; 2015.
20. Lakew Y, Bekele A, Biadgilign S. Factors influencing full immunization coverage among 12–23 months of age children in Ethiopia: evidence from the national demographic and health survey in 2011. BMC Public Health. 2015; 15(1):1–8. https://doi.org/10.1186/s12889-015-2078-6 PMID: 26224089
21. Medhanyie A, Spigt M, Kifle Y, Schaay N, Sanders D, Blanco R, et al. The role of health extension workers in improving utilization of maternal health services in rural areas in Ethiopia: a cross sectional study. BMC Health Serv Res. 2012; 12(1):1–9. https://doi.org/10.1186/1472-6963-12-352 PMID: 23043288
22. Assefa Y, Gelaw YA, Hill PS, Taye BW, Van Damme W. Community health extension program of Ethiopia, 2003–2018: successes and challenges toward universal coverage for primary healthcare services. Global Health. 2019; 15(1):1–11.
23. Rogers EM, Vaughan PW, Swalehe RM, Rao N, Svenkerud P, Sood S. Effects of an entertainment-education radio soap opera on family planning behavior in Tanzania. Stud Fam Plann. 1999; 30 (3):193–211. https://doi.org/10.1111/j.1728-4465.1999.00193.x PMID: 10546311
24. Bacha A, Mohammed K, Kuto L, Fufa D. Coffee Ceremony of the Macha Oromo in Jimma Zone, Ethiopia. International Journal. 2019; 6(1).
25. Akmatov MK, Mikolajczyk RT. Timeliness of childhood vaccinations in 31 low and middle-income countries. J Epidemiol Community Heal. 2012; 66(7):e14–e14. https://doi.org/10.1136/jech.2010.124651 PMID: 21551179
26. Mekonnen ZA, Gelaye KA, Were MC, Tilahun B. Timely completion of vaccination and its determinants among children in northwest, Ethiopia: a multilevel analysis. BMC Public Health. 2020; 20(1):1–13.
27. Okwaraji YB, Berhanu D, Persson LA. Community-based child care: household and health-facility perspectives. Dagu baseline survey, Ethiopia, December 2016-February 2017. Community-based child care Househ Heal Facil Perspect. 2017; https://www.lshtm.ac.uk/files/LSHTM-dagu-community-based-child-care.pdf
28. World Health Organization. Vaccination Coverage Cluster Surveys: Reference Manual; 2018. https://www.who.int/immunization/monitoring_surveillance/Vaccination_coverage_cluster_survey_with_annexes.pdf
29. Fleiss JL, Levin B, Paik MC. Statistical methods for rates and proportions. John Wiley & Sons; 2013.

30. Masters NB, Wagner AL, Boulton ML. Vaccination timeliness and delay in low-and middle-income countries: a systematic review of the literature, 2007–2017. Hum Vaccin Immunother. 2019; 15(12):2790–805. https://doi.org/10.1080/21645515.2019.1616503 PMID: 31070992

31. Boulton ML, Carlson BF, Wagner AL, Porth JM, Gebremeskel B, Abeje Y. Vaccination timeliness among newborns and infants in Ethiopia. PLoS One. 2019; 14(2):e0212408. https://doi.org/10.1371/journal.pone.0212408 PMID: 30779781

32. Marefiaw TA, Yenesew MA, Mihirete KM. Age-appropriate vaccination coverage and its associated factors for pentavalent 1–3 and measles vaccine doses, in northeast Ethiopia: A community-based cross-sectional study. PLoS One. 2019; 14(8):e0218470. https://doi.org/10.1371/journal.pone.0218470 PMID: 31419230

33. Geweniger A, Abbas KM. Childhood vaccination coverage and equity impact in Ethiopia by socioeconomic, geographic, maternal, and child characteristics. Vaccine. 2020; 38(20):3627–38. https://doi.org/10.1016/j.vaccine.2020.03.040 PMID: 32253099

34. Lee P. Should we adjust for a confounder if empirical and theoretical criteria yield contradictory results? A simulation study. Sci Rep 4, 6085 (2014). https://doi.org/10.1038/srep06085 PMID: 25124526

35. StataCorp. Stata Statistical Software: Release 15. College Station, TX: StataCorp LLC; 2017.

36. Hutchinson P, Lance P, Guilkey DK, Shahjahan M, Haque S. Measuring the cost-effectiveness of a national health communication program in rural Bangladesh. J Health Commun. 2006; 11(S2):91–121. https://doi.org/10.1080/10810730600974647 PMID: 17148101

37. Owais A, Hanif B, Siddiqui AR, Agha A, Zaidi AKM. Does improving maternal knowledge of vaccines impact infant immunization rates? A community-based randomized-controlled trial in Karachi, Pakistan. BMC Public Health. 2011; 11(1):1–8.

38. Murray J, Remes P, Ilboudo R, Belem M, Salouka S, Snell W, et al. The saturation+ approach to behavior change: case study of a child survival radio campaign in Burkina Faso. Glob Heal Sci Pract. 2015; 3 (4):544–56. https://doi.org/10.9745/GHSP-D-15-00049 PMID: 26681703

39. Kasteng F, Murray J, Cousens S, Sarrassat S, Steel J, Meda N, et al. Cost-effectiveness and economies of scale of a mass radio campaign to promote household life-saving practices in Burkina Faso. BMJ Global Health. 2018 3(4):e000809. https://doi.org/10.1136/bmjgh-2018-000809 PMID: 30057798

40. Kang H. The prevention and handling of the missing data. Korean J Anesthesiol. 2013; 64(5):402–6. https://doi.org/10.4097/kjae.2013.64.5.402 PMID: 23741561

41. Miles M, Ryman TK, Dietz V, Zell E, Luman ET. Validity of vaccination cards and parental recall to estimate vaccination coverage: a systematic review of the literature. Vaccine. 2013; 31(12):1560–8. https://doi.org/10.1016/j.vaccine.2012.10.089 PMID: 23196207

42. Central Statistical Agency (CSA)[Ethiopia] and ICF. Ethiopia Demographic and Health Survey, Addis Ababa. Cent Stat Agency. 2016; Addis Ababa, Ethiopia, and Rockville, Maryland, USA: CSA and ICF. https://dhsprogram.com/pubs/pdf/FR328/FR328.pdf
